# 

**Published:** 1893-07-15

**Authors:** 


**OUR NEW OFFICES**

428, Strand, W.C., will henceforth be the Office of this Journal.

**Notices of Births, Deaths, and Marriages,** are inserted in this column at a charge of 2s. 6d. for an announcement not exceeding four lines.

### NOTICE TO CORRESPONDENTS.

All MS., Letters, Books for Review, and other matters intended for the Editor should be addressed The Editor, The Lodge, Porchester Square, London, W.

The Editor cannot undertake to return rejected M.S., even when accompanied by stamped directed envelope.

**Notice to Advertisers and Subscribers.**

All Advertisements, Orders for Copies of The Hospital, and Business Communications should be addressed to The Publisher, *not to the Editor*, The Hospital, 428, Strand, W.C.

The Cost of Subscription, post free, is as follows:—Per week, 2½d.; per quarter, 2s. 6d.; per half-year, 5s.; per annum, 8s. 6d. Foreign:—Per annum, 10s. 8d.; payable, in all cases, in advance.

**"Burdett's Hospital Annual," 1893.**

Fourth year of publication. 700 pp. Boards, gilt lettering. A most complete guide to British, Colonial, Indian, and American Hospitals, Dispensaries, Nursing and Convalescent Institutions, and Asylums. Price 5s. Published by The Scientific Press (Limited), 428, Strand, W.C.

**NOTICE.—The Index for Vol. XIII. (ending March 1893) is on sale at the Office of this Journal, price 2½d., post free.**

### SCRAPS AND GLEANINGS.

It is said that the drought has already cost the War Office £70,000 for extra forage for the horses.

The Honiton Town Council are taking steps for the provision of a new hospital for infectious diseases.

The contents of 198 purses placed on the foundation-stone of the new infirmary at Halifax amounted to £717 14s.

The Fellows of the Royal Geographical Society have, by special resolution, refused to admit ladies as honorary members.

The late Mr. Albert Levy has bequeathed £200 to the Margate Cottage Hospital, and £200 to the Royal Sea Bathing Infirmary, Margate.

Mrs. Gladstone gave a concert at Carlton House Terrace last week in aid of the Hawarden Orphanage and Notting Hill Training School

The Free Eye and Ear Hospital, Southampton, has been offered a site in Grosvenor Square provided that a building worth at least £2,000 is erected thereon.

At the monthly meeting in June of the directors of the Longton Cottage Hospital the financial statement showed a considerable falling off in subscriptions.

Important sanitary improvements are contemplated at the South Charitable Infirmary, Cork, for the efficient carrying out of which funds are urgently needed.

A hospital demonstration took place at Richmond on June 24th, in connection with the Royal Hospital. Nearly £80 was counted the same night, of which sum £37 was in coppers.

A garden party was held at the Botanical Gardens, Old Trafford, on June 23rd, and the proceeds were handed over to the managers of the Sick Children's Hospital at Pendlebury.

In 1863 the proportion to the general population of those receiving poor-law relief was highest, being 49·7 per 1,000 of the population. In the present year it has gone down to 22·9.

A fifth part of the territory was affected by typhus in the recent epidemic in France. The first case occurred in December, and tramps and vagabonds were the chief carriers of the disease.

Two houses in the centre of the parish of Marylebone have been placed by the generous gift of Lord Portman at the disposal of the sanitary authorities for the accommodation of scarlet fever patients.

A summer fête and garden party was given at Earlswood Asylum, on Wednesday last. Plenty of amusement was provided for the guests, the entertainment concluding with a brilliant race of fire-balloons.

The total amount collected in Southampton on Hospital Sunday this year amounted to £1,1[illegible]1 10s. 1d. The deduction of expenses leaves a net sum of £1,105 15s. 10d., as against £1,077 2s. 11d. last year.

The new central block of the North London Hospital for Consumption at Hampstead was opened on Tuesday by the Princess Christian. Purses to the value of £175 were presented by ladies and children.

The remonstrances of the inhabitants of Lewisham have proved unavailing, and the Local Government Board has consented to the erection of a new fever hospital for London on the proposed site at Hither Green.

The small-pox hospital at Aubervilliers, near Paris, was recently struck by lightning and set on fire. It consisted of five wooden buildings, and the patients were removed with considerable risk and difficulty.

### Books Received.

Smith, Elder, and Co..

"Hypnotism, Mesmerism, and the New Witchcraft." By Ernest Hart.

Simpkin, Marshall, and Co.

"Domestic Economy for Students and Teachers." By Ada Colley.

Methuen and Co.

"A Change of Air." By Anthony Hope.

Swan Sonnenschein and Co.

"Beauty and Hygiene for Women and Girls." By a Specialist.

Reeves and Turner.

"The Law of Cremation." By Aubrey Richardson, Solicitor.

Digby, Long, and Co.

"The Princess's Private Secretary." From the Italian of A. Barrili. By His Honour Justice Stephen.

Joseph Hughes and Co..

"Practical Kindergarten Lessons for English Infant Schools." By Mrs. Mortimer.

**Periodicals and Pamphlets.**—How to Feed Babies and Invalids, Edited by G. Mellin; The Cornhill Magazine; The Young Gentlewoman; Forward.

### The Hospital Advertiser.

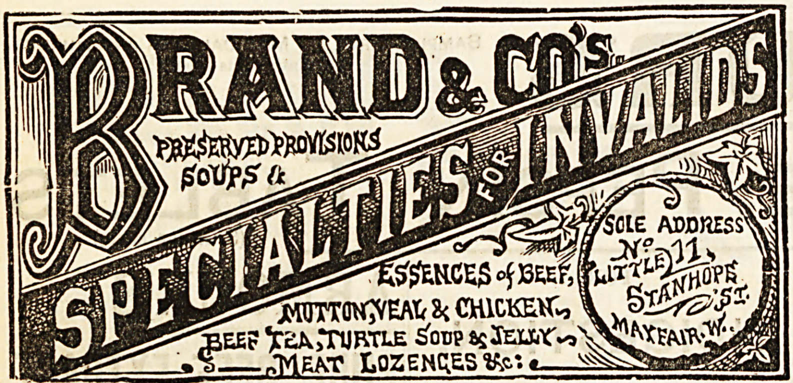

**BRAND & Co.'S MUTTON AND CHICKEN JELLIES For INVALIDS.**

MAYFAIR, W.

**PETER MÖLLER'S COD LIVER OIL**

(*By Patented Process.*)

A small sample can be had free from the London Office (or by post 3d.), 43, Snow Hill, E.C.

BEST AND CHEAPEST.

ESTABLISHED 1825.

**NEAVE'S FOOD**

FOR INFANTS, CHILDREN, INVALIDS, AND THE AGED.

In Patent Air-Tight Tins.

### MEDICAL VACANCIES AND APPOINTMENTS.

### APPOINTMENTS.

W. J. T. Barker, L.R.C.P.Lond., M.R.C.S.Eng., appointed Medical Officer for the Second District of Streatham of the Wandsworth and Clapham Union. J. Beattie, L.R.C.P., L.R.C.S.Edin., L.F.P.S.Glasg., appointed Medical Officer for the Staindrop District of the Teesdale Union. W. Bedell, appointed Assistant Medical Officer to the Infirmary of the Parish of St. Leonard Shoreditch. M. F. Cahill, L.R.C.P., L.R.C.S.I., appointed Assistant Physician to the Children's Hospital, Dublin. C. J. Cressy, L.R.C.P., M.R.C.S., appointed Medical Officer for the Third District of the Highworth and Swindon Union. L. W. Dryland, M.R.C.S.Eng., L.R.C.P.Lond., appointed House Surgeon to the Northampton General Infirmary. W. McAdam Eccles, M.B., B.S.Lond , F.R.C.S.Eng., appointed Assistant Surgeon to the West London Hospital. C. J. Fuller, L.R.C.P.Lond., M.R.C.S.Eng., appointed Medical Officer for the East Woolwich District of the Woolwich Union. M. R. Gooding, L.R.C.P.Lond., M.R.C.S.Eng., appointed Medical Officer of Health to the Bideford Local Board. W. H. Gossage, M.R.C.S.Eng., L.R.C.P.Lond., appointed House-Surgeon to the Westminster Hospital. F. Grant, L.R.C.P.Edin., M.R.C.S.Eng., reappointed Medical Officer of Health for the Market Harborough Urban Sanitary District. C. B. Gratte, L.R.C.P.Lond., M.R.C.S.Eng., reappointed Medical Officer for the Marshfield District of the Newport Union. G. Hall, M.D.Edin., reappointed Medical Officer of Health for the Hambledon Union. H. Harris, L.R.C.P.Edin., M.R.C.S.Eng., appointed Medical Officer for the Brandwood District of the Rochdale Union. C. H. Haycroft, M.R.C.S.Eng., L.S.A., reappointed Medical Officer for the Colebrook and Coldridge District of the Crediton Union. H. Hollis, M.B., B.C.Camb., appointed Assistant House Surgeon to the Northampton General Infirmary. J. A. Hutchinson, M.D.Durh., M.R.C.S.Eng., appointed Medical Officer of Health for the Northallerton Urban Sanitary District. R. W. Jamie, M.B., C.M.Edin., appointed Medical Officer for the No. 5 District of the Ashby de la Zouche Union. G. H. Johnston, L.R.C.P., L.R.C.S.Edin., appointed Junior Assistant Medical Officer to the North Riding Lunatic Asylum, Clifton, York. H. W. Kershaw, M.R.C.S., L.R.C.P., appointed Senior Assistant Medical Officer to the North Riding Lunatic Asylum, Clifton, York. W. Livesay, M.D.Edin., appointed Medical Officer for the Sudbury District of the Uttoxeter Union. D. R. McArthur, M.D.Edin., appointed Medical Officer for the East District of the Luton Union. J. McNidder, M.B., C.M.Edin., appointed Visiting Surgeon to the Hull and Sculcoates Dispensary. R. Martin, L.R.C.P., L.R.C.S.Edin., L.F.P. and S.Glasg., appointed Medical Officer to the Banford Sick Fund Society. G. H. Moorhead, appointed Medical Officer of Health for Hunsworth Rural Sanitary District. T. H. Morse, F.R.C.S.Eng., L.R.C.P. Edin., appointed Honorary Medical Officer to the Cromer Cottage Hospital. J. Morton, M.B.Lond., M.R.C.S.Eng., reappointed Medical Officer of Health for the Guildford Urban Sanitary District. J. B. Moxon, M.R.C.S.Eng., L.S.A., reappointed Medical Officer of Health to the Brigg Rural Sanitary Authority. J. R. S. Nodes, L.R.C.P.Lond., M.R.C.S.Eng., appointed Medical Officer for the No. 2 (Worthing) District of the East Preston Union. E. C. Osborn, L.R.C.P.Lond., appointed Senior House Surgeon to the Westminster Hospital. H. E. Owen, L.R.C.P., appointed Medical Officer of Health for the Knightsbridge District Local Board. C. E. Perry, M.D.Brux., L.R.C.P.Edin., reappointed Medical Officer of Health to the Sandgate Local Board. G. H. Shaw, M.R.C.S.Eng., appointed Honorary Secretary of the Sheffield General Infirmary. T. W. Shortridge, M.D.Brux., L.R.C.P., L.R.C.S.Edin., reappointed Medical Officer of Health for Honiton. W. W. Stocks, M.R.C.S., L.R.C.P.Lond., appointed Medical Officer for the Second District of the Hendon Union. R. H. S. Spicer, M.D.St. And., L.R.C.P.Edin., M.R.C.S.Eng., reappointed Medical Officer for the East and West Anstey Districts of the South Molton Union. S. Spokes, M.R.C.S., L.D.S., appointed Dental Surgeon to University College Hospital. F. Stockwell, M.D.Lon., M.R.C.S.Eng., reappointed Medical Officer of Health for Sick Wincanton Rural Sanitary District. A. F. Voelcker, M.D.Lond., appointed an Assistant Physician to the Hospital for the Children, Great Ormond Street. E. C. Warren, L.R.C.S.Edin., L.M., L.S.A., reappointed Medical Officer of Health to the Gillingham Local Board. H. W. Williams, M.D. (late Physician), ap-

**CHARING CROSS HOSPITAL.**

Salary £50 per annum, with Board, Lodging on the premises, and Washing.

**MEDICAL VACANCIES AND APPOINTMENTS—(*continued*)**

pointed Consulting Physician to the Infirmary for Consumption and Diseases of the Chest and Throat, Margaret Street, W. A. M. Whitestone, M.B.Dub., B.Ch., appointed Assistant Medical Officer to the Hollingbourne Union. W. B. Winckworth, M.R.C.S.Eng., L.R.C.P.Lond., appointed House Physician to the Westminster Hospital. E. G. Younger, M.D.Brux., M.R.C.P.Lond., late Visiting Physician, appointed Physician to the Infirmary for Consumption and Diseases of the Chest and Throat, Margaret Street, W. Mr. A. H. Gordon, B.A., M.B., and B.C., St. John's, Cambridge, and of Guy's Hospital, has been appointed House Surgeon to the Croydon General Hospital.

### VACANCIES.

The following vacancies are announced this week, the amount of salary in each case being given after the name of the institution:

**Resident Medical Officers.**

General Infirmary, Leeds. Resident Medical Officer. Salary £100 per annum, with board, washing, and lodging. Applications to Mr. Littlewood, Secretary to the Faculty, Infirmary, Leeds, by July 21st.

Liverpool Dispensaries. Assistant Surgeon; unmarried. Salary £80 per annum, with apartments, board, and attendance. Applications to R. R. Greene, Secretary, by July 24th.

Manchester Royal Infirmary. Resident Medical Officer, not less than 25 years of age and unmarried; doubly qualified. Appointment for one year, but holder of office may be re-elected. Salary £150 per annum, with board and residence. Applications to the Chairman of the Board by July 29th.

Stafford General Infirmary, Stafford. Assistant House Surgeon. Board and residence provided. Applications to the House Surgeon by July 20th.

Wirral Children's Hospital, Woodchurch Road, Birkenhead. Resident House Surgeon. Salary £50 per annum, with board, lodging on the premises, and washing. Applications to P. W. Atkin, Honorary Secretary, 25, Lord Street, Liverpool, by July 19th.

**Non-Resident Medical Officers.**

Caerphilly Local Board. Medical Officer of Health. Salary £40 per annum. Applications to David Lewis, Clerk to the Board, Caerphilly, by July 19th.

Charing Cross Hospital, W.C. Assistant Surgeon; must be F.R.C.S. Eng. Applications to the Chairman of Committee by July 24th.

Chelsea Hospital for Women, Fulham Road, S.W. Lady Dispenser. Salary £60 per annum. Applications, on forms to be obtained at the Hospital, to the Secretary by July 17th.

Hospital for Epilepsy and Paralysis, 32, Portland Terrace, Regent's Park, N.W. Physician. Applications to the Secretary, H. Howgrave Graham, by July 15th.

The Yorkshire College, Leeds. Demonstrator of Anatomy. Particulars from the Registrar.

University College, London. Assistant Obstetric Physician. Applications to J. M. Horsburgh, M.A., Secretary, by July 18th.

### PASS LIST.

**University of Cambridge.**

EXAMINATIONS FOR MEDICAL AND SURGICAL DEGREES, EASTER TERM, 1893.

Second Examination.

Part I. Pharmaceutical Chemistry.—Examined and approved: Abercrombie, Gonv. and Cai.; Allfrey, Trin.; Baker, M.A.; Boulton, Cla.; Bradford, Emm.; F. H. Burton, Cla.; Butler, Joh.; Chapman, B.A., Corp. Chr.; Corner, Sid. Suss.; Daniel, Emm.; A. H. Donaldson, B.A., Gonv. and Cai.; Ellis. Gonv. and Cai.; Glasier, Emm.; Golby, Joh.; Gostling, Gonv. and Cai.; Gregory, Joh.; Halahan, B.A., Corp. Chr.; Hale, Chr.; Hunt, Trin.; J. Lea, B.A., Gonv. and Cai.; Levick, Jes.; Lobb, B.A., Gonv. and Cai.; F. McCleary, B.A., Trin. H.; E. B. Marriott, B.A., Cla.; Nix, B.A., Pemb.; Parsons, B.A., Corp. Chr.; Porter, Emm.; Powell, Emm.; Rose, B.A., Joh.; P. W. G. Sargent, Joh.; Hon. G. H. Scott, B.A., Trin.; Shewell, Trin.; Tallent, Joh.; Troup, B.A., Pemb.; Tucker, Gonv. and Cai.; E. A. Wilson, Gonv. and Cai.; Yeld, Trin.

Part II. Human Anatomy and Physiology.—Examined and approved: J. L. Allen, B.A., King's; Biss, B.A., King's; Cornaby, B.A., Non Coll.; Dashwood, B.A., Magd.; Eardley, M.A., Joh.; G. L. Edwards, B.A., Trin.; Gaine, Emm.; L. S. Gaskell, B.A., Chr.; F. A. Godson, B.A., Joh.; Grummitt, B.A., Gonv. and Cai.; Haslam. B.A., Gonv. and Cai.; Hayward, B.A., Gonv. and Cai.; Heywood, Emm.; Higgins, B.A., Non Coll.; Holmes, Joh.; Hyde, B.A., Cla.; Lance, B.A., King's; Lillie, Joh.; McDougall, Joh.; Macnamara, B.A., Pet.; Manners-Smith, B.A., Down; Maturin, B.A., Gonv. and Cai.; May, B.A., Cla.; Mills, B.A., Cla.; Molesworth, Gonv. and Cai.; Muir, B.A., King's; Muriel, Down; Newstead, B.A., Chr.; Nix, B.A., Pemb.; Parker, Gonv. and Cai.; Pearce, B.A., Trin.; Pearse, B.A. Trin.; Penny, B.A., Pemb.; Pentreath, B.A., Queens'; Prince, Gonv. and Cai.; Hon. G. H. Scott, B.A., Trin.; Sing, B.A., Chr.; M. H. Smith, B.A., Pet., Somers, B.A., Pemb.; Stead, B.A., Gonv. and Cai.; K. S. Storrs, Emm.; Sturrock, B.A., Gonv. and Cai.; Turner, B.A., Emm.; Twigg, B.A., Chr.; Tyson, B.A., Gonv. and Cai.; A. B. Ward, B.A., H. Selw.; Wills, B.A., Gonv. and Cai.; Woodhouse, B.A., King's; Woolley B.A., Chr.; Worthington, B.A., Pemb.

